# Thermal Degradation and Combustion Behaviors of Polyethylene/Alumina Trihydrate/Graphene Nanoplatelets

**DOI:** 10.3390/polym11050772

**Published:** 2019-05-01

**Authors:** Chunfeng Wang, Jihua Wang, Zhenlong Men, Yongliang Wang, Zhidong Han

**Affiliations:** 1 School of Materials Science and Engineering, Harbin University of Science and Technology, Harbin 150040, China; iwsaml@163.com (C.W.); wjh99924@126.com (J.W.); 15004652903@163.com (Z.M.); yongliangwang@hrbust.edu.cn (Y.W.); 2 Key Laboratory of Engineering Dielectrics and Its Application, Ministry of Education, Harbin University of Science and Technolog, Harbin 150080, China

**Keywords:** graphene nanoplatelets, polyethylene, alumina trihydrate, flame retardancy, cone calorimeter

## Abstract

Graphene nanoplatelets (GNPs) were prepared from expanded graphite (EG) with fully exfoliated structure via ball milling coupled with ultrasonication. The structure of multi-layered GNPs was characterized with transmission electron microscopy (TEM), scanning electron microscopy (SEM), X-ray diffraction (XRD), atomic force microscopy (AFM) and Raman spectroscopy. By compounding alumina trihydrate (ATH) with GNPs, the well dispersed mixture of ATH/GNP was obtained, and it showed high flame retardant effectiveness in polyethylene (PE). The peak heat release rate (peak-HRR) decreased by 20% was proven by a cone calorimeter with the addition of GNPs as low as 0.2 wt % in PE/ATH. The results of thermogravimetric analysis (TG) illustrated the improved thermal stability and lower weight loss rate of PE/ATH/GNP than PE/ATH. A protective char with GNPs was evidenced by SEM and X-ray photoelectron spectroscopy (XPS). The well exfoliated structure and good dispersion of GNPs accounted for the formation of effective barrier, which made a profound contribution to the enhanced flame retardancy.

## 1. Introduction

Concerns about fire safety and environment protection give rise to increasing demands for environmental-friendly flame-retardant materials [1,2]. As one of the most extensively applied thermoplastics in wire and cable industry, polyethylene (PE) and its flame retardancy have attracted much attention [3,4,5]. Alumina trihydrate (ATH) is commonly used as flame retardant for PE in obtaining low smoke halogen-free flame retardant (LSHFFR) compounds for wire and cables [6,7]. Even though LSHFFR compounds have been widely applied [8], challenges have to be faced due to the limited flame-retardant effectiveness of ATH. Many efforts have been made to enhance the flame retardancy of ATH based compounds [9,10,11,12,13,14]. 

By using nanoparticles, the flame-retardant properties can be greatly improved [15]. One of the outstanding characteristics of nanoparticles is that they can significantly reduce the heat release rate (HRR) at quite low loadings, less than 5 wt % for clays [16]. Nanocarbon materials such as nanotubes and nanofibers were widely investigated as flame retardant additives for polymers, fire-retardant pads and coatings [17,18,19]. Graphene nanoplatelets (GNPs) were also found effective in improving the flame retardant properties of the polymers [20,21,22]. For poly(vinyl alcohol) (PVA), the flame retardant efficiency of GNPs was reported to surpass that of both Na-montmorillonite (MMT) and multiwall nanotubes (MWNTs) with the same content [23]. Well-exfoliated GNPs were revealed to be preferable to less exfoliated GNPs, micron-sized expanded graphite (EG) or carbon black (CB) and MWNTs in flame retardant isotactic polypropylene (PP) [24]. GNPs showed good flame retardant effects when used together with melamine polyphosphate (MPP) [25], organically modified MMT [26], or intumescent flame retardant (IFR) [27,28]. 

Graphite was considered as one of the abundant natural sources to prepare graphene. GNPs, typically derived from expanded graphite (EG) and graphite oxide (GO), have advantages in flame retardant applications [29,30,31]. Thus, attempts were made to find a massive and facile way for preparing GNPs as flame retardants [32,33]. For instance, an electrochemical route was reported to prepare multifunctional graphene-based flame retardants with phosphazene rings, by which the exfoliation and functionalization of graphene were achieved simultaneously [34]. Recently, the effects of the dispersion state of graphene on the flammability and fire behaviors of IFR composites were reported [35]. The flame-retardant performance of GNPs showed a strong dependence on the dispersion in the matrix as well as their thickness and lateral dimensions. 

The enhanced fire retardancy of PE/ATH composites with GNPs was reported by our group [36]. Hereinafter, GNPs were prepared from EG and used in flame retardant PE/ATH composites for LSHFFR applications. A facile way was proposed to prepare ATH/GNP as halogen-free flame retardant by compounding ATH with GNP suspension. The thermal degradation and combustion behaviors of PE/ATH/GNP were investigated to reveal the flame-retardant mechanism for the enhanced fire retardancy at a loading as low as 0.2 wt %. 

## 2. Materials and Methods

### 2.1. Materials

Polyethylene (PE, Q210) was purchased from China Petroleum & Chemical Corporation (Sinopec, Shanghai, China) with a melt flow rate of 2.1 g/10 min and a density of 0.921 g/cm^3^. Alumina trihydrate (ATH, HF-1) with an average particle size of 2 μm was kindly provided by Shandong Alunimum Company of Aluminum Corporation of China (Chinalco). Natural flaky graphite (LC50-99.9) with a particle size of 50 mesh and a purity of 99.9% was purchased from Tianheda Graphite Co. Ltd. (Qingdao, China). The other reagents were purchased from Sinopharm Chemical Reagent Co. ltd. (Beijing, China) and used without further treatment. 

### 2.2. Preparation of GNPs

Graphene nanoplatelets (GNPs) were prepared from expanded graphite (EG) via mechanical milling coupled with ultrasonication. Firstly, expandable graphite (EDG) was prepared from natural graphite (NG). 10 g NG was immersed in a solution of 30 mL sulfuric acid (98%), 10 g sodium nitrate and 1 g potassium dichromate. The reaction was progressed at 30 °C for 30 min and the product was fully washed with distilled water to a pH value about 7. EDG was obtained after the product was dried at 60 °C in a vacuum oven for 6 h. EG was collected by thermally treating EDG for 1 min in a muffle oven preheated to 800 °C. The as-prepared EG was put in sodium dodecyl sulfate (SDS) solution and treated by ball milling for 2 h. The obtained solution was ultrasonically treated for 4 h to obtain a suspension of GNPs.

### 2.3. Preparation of ATH/GNP

ATH was mixed with the suspension of GNPs and ground by ball milling for 2 h. The mixed suspension was dried at 100 °C for 8 h and ground again for 1 h to obtain the mixture of ATH and GNPs (ATH/GNP). To get well exfoliated GNPs, 0.1 g EG was dispersed in 100 mL water to get a suspension with 1 mg/mL GNPs. And 19.9 g ATH was added into the suspension to collect 20.0 g mixture of ATH/GNP. The flame-retardant mixture was thus prepared at the ATH/GNP mass ratio of 100/0.5.

### 2.4. Preparation of Flame-Retardant Composites

The composites of PE/ATH/GNP were prepared by melt blending in a RM-200C mixer rheometer (Hapro Harbin Electric Technology Co. Ltd., Harbin, China) at 160 °C with a speed of 60 rpm. PE was added into rheometer to melt, and then ATH or ATH/GNP was introduced into the mixing chamber and blended with PE melt to a torque balance. Then, the blending proceeded for 5 min and the composites were obtained. The loading of ATH or ATH/GNP in the composites was 40 wt %, respectively. Two composites were prepared, PE/ATH (60% PE and 40% ATH) and PE/ATH/GNP (60.0% PE, 39.8% ATH and 0.2% GNPs). Specimens for testing were prepared by compression molding.

### 2.5. Characterization

Transmission electron microscopy (JEM-2100, JEOL, Tokyo, Japan) was used to observe the morphology of GNPs at an accelerator voltage of 200 kV by depositing the suspension of GNPs on 200 mesh Cu grid. Scanning electron microscopy (Sirion200, Philips, Eindhoven, the Netherlands) was used to observe the morphology of ATH and ATH/GNP after the samples were metallized with gold. X-ray diffraction (X′Pert PRO, PANalytical, Almelo, the Netherlands) was conducted at 40 kV with 40 mA current in reflection mode with Cu Kα. Atomic force microscopy (Dimension Icon, Bruker AXS, Madison, WI, USA) was conducted in tapping mode by casting GNPs on a silicon wafer. Thermogravimetric analysis (STA600, PerkinElmer, Norwalk, CT, USA) was carried out at a heating rate of 20 °C/min in nitrogen flow. Raman spectroscopy (HR 800, HORIBA Jobin Yvon, Villeneuved’Ascq, France) was carried out at 457.9 nm with an Ar ion laser beam. X-ray photoelectron spectroscopy (PHI Quantera II, Ulvac-PHI, Kanagawa, Japan) was conducted with monochromatic Al-Kα. All binding energies were referenced to the adventitious C1s at 284.6 eV. The vacuum of the main vacuum chamber is better than 10^−7^ Pa. The fire testing was performed on the samples with the size of 100 mm × 100 mm × 3 mm at a heat flux of 35 kW/m^2^ in the horizontal configuration according to ISO 5660 by using an FTT cone calorimeter (CONE). The morphological structure of the residual char after CONE testing was characterized by SEM.

## 3. Results

### 3.1. Morphology of GNPs

**Figure 1:**
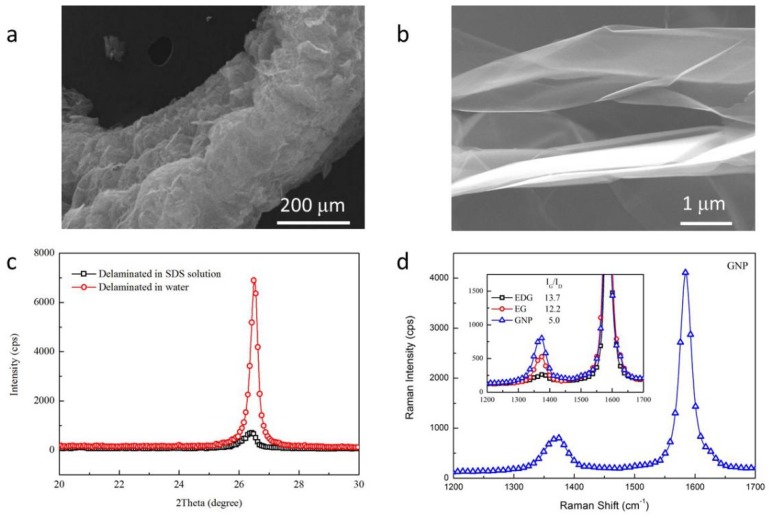
(**a**,**b**) SEM micrographs of EG, (**c**) XRD patterns of delaminated products of EG in water and SDS solution, (**d**) Raman spectrum of GNPs with inserted spectra of EDG, EG and GNPs.

EDG was thermally treated at 800 °C to get worm-like EG as shown in Figure 1a. Thanks to the well intercalated structure of EDG [37], fully exfoliated nanoplatelets were observed in Figure 1b and linked in the network of EG worm. According to the sharp decrease of diffraction intensity in Figure 1c, the delamination of the interlinked layers was proved to be efficient in the solution of SDS than that in water. Compared with the strong oxidation conditions in preparing graphite oxide [38], EDG was prepared from NG under the mild conditions, which made it possible for the high I_G_/I_D_ value in the Raman spectra of EDG and EG. Although the decrease of I_G_/I_D_ value was found in comparison of GNPs with EG, the I_G_/I_D_ value in the Raman spectrum of GNPs in Figure 1d was still remarkable for the GNPs prepared from NG. 

**Figure 2:**
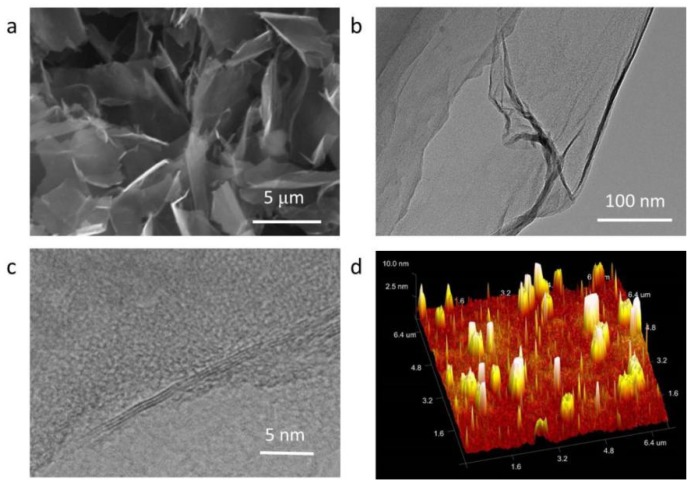
(**a**) SEM, (**b**,**c**) TEM, and (**d**) AFM micrographs of GNPs.

The EG concentration of 1 mg/mL was adopted to obtain the suspensions of GNPs. Figure 2 showed the SEM and TEM micrographs of GNPs, which presented well delaminated nanoplatelets with large lateral size of around 2 μm. GNPs were found very thin and some stacked nanoplatelets were observed in Figure 2b. Three-layered GNPs, as shown in Figure 2c, was also obtained in a mixture of multi-layered GNPs as a result of the AFM profile in Figure 2d. Accordingly, EG with fully exfoliated structure would be a good candidate to prepare few-layered GNPs with high structural integrity. 

### 3.2. Morphology of ATH/GNP

**Figure 3:**
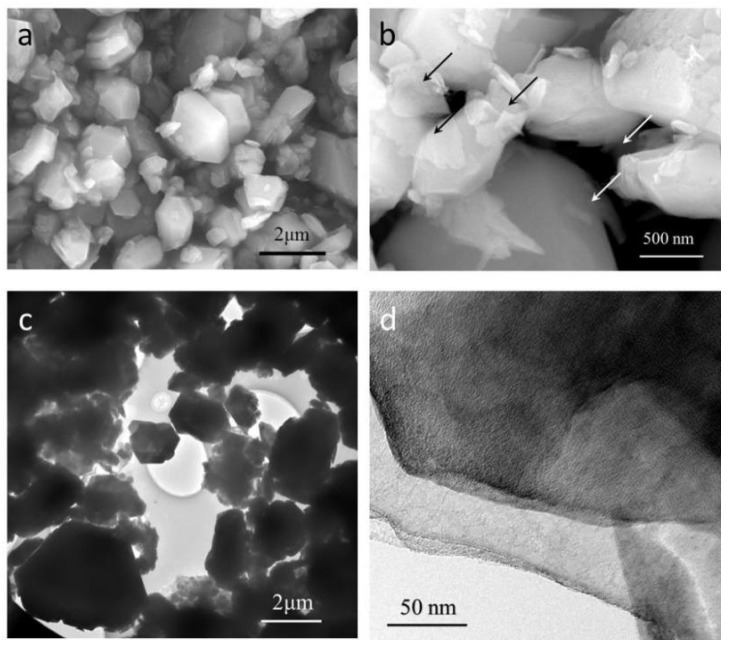
SEM micrographs of (**a**) ATH and (**b**) ATH/GNP; TEM micrographs of (**c**,**d**) ATH/GNP.

After mixed with ATH, GNPs were found well dispersed with ATH according to the SEM and TEM micrographs in Figure 3. The nearly transparent nanoplatelets as well as their thin thickness evidenced the well delaminated structure of GNPs, which were pointed by the arrows in Figure 3b. The morphology of ATH in ATH/GNP (shown in Figure 3b) seemed similar to that of pristine ATH in Figure 3a. Some nanoplatelets were found stacked in ATH/GNP by TEM micrograph in Figure 3d. 

### 3.3. Thermal Degradation of ATH/GNP

**Figure 4:**
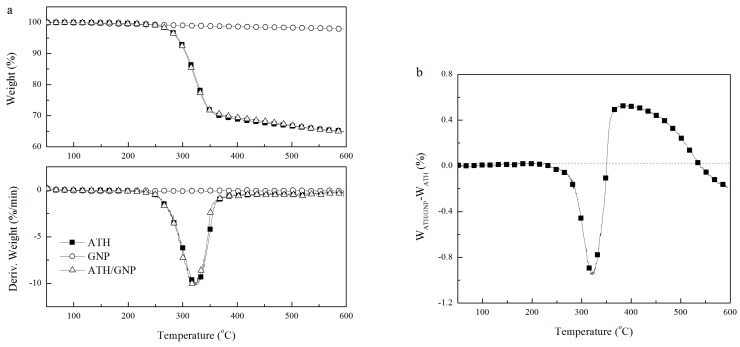
(**a**) TG curves of GNPs, ATH and ATH/GNP; (**b**) TG weight difference between ATH/GNP and ATH.

Figure 4 showed the TG curves of GNPs, ATH and ATH/GNP. GNPs had good thermal stability in nitrogen atmosphere with only 2% weight loss at 600 °C. ATH started thermal decomposition at about 250 °C and gave about 35% weight loss at 600 °C due to the release of water. The dehydration process of ATH ranged from 250 °C to 360 °C with the maximal weight loss rate at 323 °C. The TG/DTG curves of ATH/GNP with 0.5% GNPs in Figure 4a were found similar to that of ATH. Both ATH and ATH/GNP presented the similar maximal weight loss rate at 323 °C. 

By comparing the weight difference between ATH/GNP and ATH in Figure 4b, ATH/GNP showed more weight loss in the temperature range from 250 °C to 360 °C and left more char in the temperature range from 360 °C to 550 °C. Accordingly, the thermal decomposition of ATH/GNP initiated at lower temperature in comparison with ATH. The TG data were collected in Table 1. The temperature corresponding to 10% weight loss (T_10_ in Table 1) of ATH/GNP (305 °C) was 2 °C lower than that of ATH (307 °C). When the dehydration completes, ATH/GNP shows better thermal stability. The temperature at 30% weight loss (T_30_ in Table 1) of ATH/GNP was 13 °C higher than that of ATH. 

### 3.4. Thermal Degradation of PE/ATH/GNP

**Figure 5:**
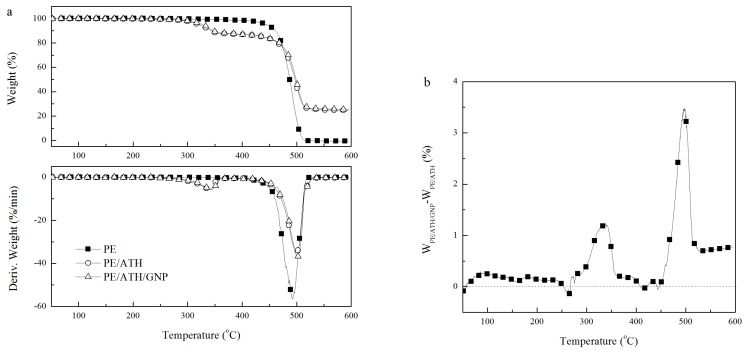
(**a**) TG curves of PE, PE/ATH and PE/ATH/GNP; (**b**) TG weight difference between PE/ATH/GNP and PE/ATH.

Figure 5 showed the TG/DTG curves of PE, PE/ATH and PE/ATH/GNP and Table 2 illustrated the TG results. PE degraded completely with no residue at 600 °C. Both PE/ATH and PE/ATH/GNP presented a degradation peak from 250 °C to 360 °C due to the dehydration of ATH. As a result, PE/ATH and PE/ATH/GNP showed the lower temperature at 5% weight loss (T_5_ in Table 2) than PE. Furthermore, PE/ATH/GNP had better thermal stability than PE/ATH by showing a 7 °C higher T_5_. When the dehydration of ATH completed, the char would act as a barrier to stabilize the PE chains, which led to a lower degradation rate at higher temperature. By comparing the weight difference between PE/ATH/GNP and PE/ATH in Figure 5b, PE/ATH/GNP showed less weight loss during the degradation process and more char residue at 600 °C, which indicated the charring effect of GNPs.

### 3.5. Combustion Behaviors

**Figure 6:**
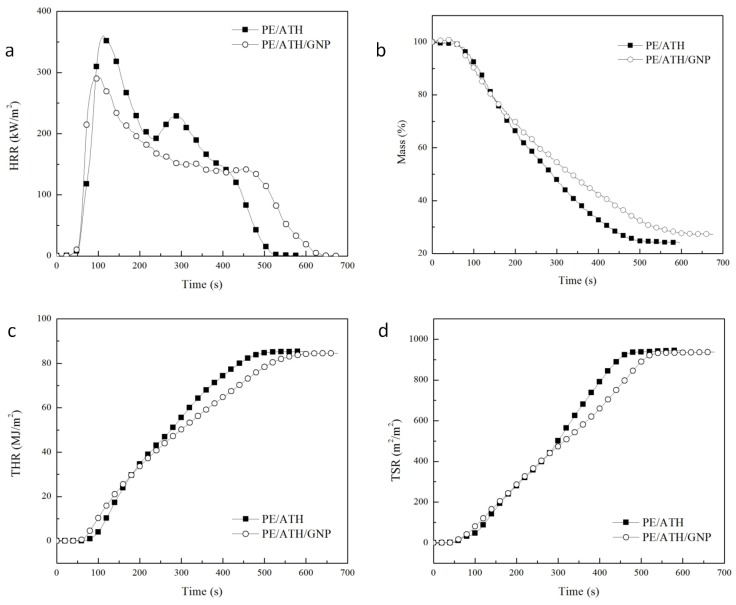
(**a**) HRR, (**b**) Mass, (**c**) THR and (**d**) TSR curves of PE/ATH and PE/ATH/GNP by Cone testing.

Cone calorimeter was applied to determine the heat release rate (HRR), total heat release (THR) and other combustion characteristics of wire and cable compounds under forced flaming conditions. To determine the effect of GNPs on the combustion behaviors of PE/ATH, the curves of HRR, THR, Mass and total smoke release (TSR) were collected and shown in Figure 6. The HRR curve of PE/ATH showed two distinct peaks. In comparison to PE/ATH, the peak-HRR value of PE/ATH/GNP decreased by 20% with only 0.2 wt % GNPs addition. Furthermore, the HRR value of the second peak was reduced by 38%, and the peak gap between two HRR peaks enlarged from 177 s to 361 s, indicating the formation of effective barrier with addition of GNPs.

The reduced HRR can be attributed to the formation of a protective char during the combustion testing [21,24,36]. For PE/ATH/GNP, the barrier effect of the char was enhanced by GNPs. Accordingly, the mass loss, THR and TSR were all suppressed once the barrier forms during combustion, which could be obviously observed from the curves in Figure 6b–d. The intersection point of the two curved indicated the formation of the char and its effectiveness in restraining the heat and smoke release. Even though the THR and TSR values for PE/ATH and PE/ATH/GNP at the end of the testing were similar, the time for PE/ATH/GNP to reach the value was approximate 100 s longer. Such results revealed the improved barrier effect of the char in suppressing the heat and mass transfer.

### 3.6. Char Morphology and Structure

**Figure 7:**
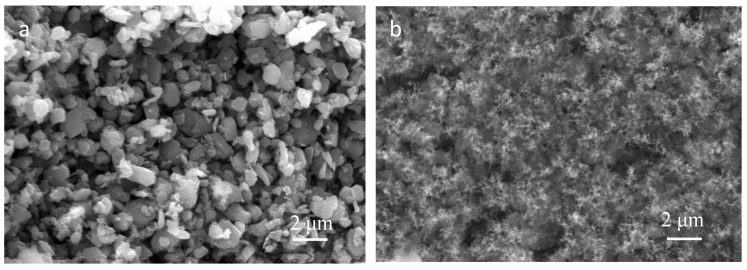
SEM micrographs of surface char residue of (**a**) PE/ATH and (**b**) PE/ATH/GNP after Cone testing.

The char morphology of PE/ATH/GNP and PE/ATH was shown in Figure 7. The residues of PE/ATH were mainly composed of alumina formed due to the decomposition of ATH. Thanks to the addition of GNPs, the residue of PE/ATH/GNP was of profound difference with that of PE/ATH. The surface of PE/ATH/GNP was covered by a fluffy and well continuous layer of char, which was in great contrast to the discrete char layer of PE/ATH. 

The element analysis on the surface char was investigated by XPS and the results were presented in Table 3. The char of PE/ATH and PE/ATH/GNP was composed of C, Al and O. The high atomic ratios of C:O and C:Al for PE/ATH/GNP char revealed the carbonaceous characteristics with addition of GNPs, which was different from that of PE/ATH. Meanwhile, the binding energy of C1s in PE/ATH and PE/ATH/GNP was 285.4 eV and 284.6 eV, respectively, which indicated that a conductive char formed, and the accumulated charge could be released. The char of PE/ATH/GNP was mainly composed of graphene-structured carbon in sp^2^ hybridized bonding while that of PE/ATH was in graphite-like structure [38]. 

## 4. Discussion

Well delaminated GNPs can be prepared by mechanical milling coupled with ultrasonication, and the suspension with high GNPs concentration of 1 mg/mL was obtained. By removing the water of suspension, GNPs can be obtained and used to prepare polymer-based composites. However, the aggregation of GNPs and stacking of the nanoplatelets would potentially make it difficult to disperse in the PE matrix, which may be detrimental to the composite properties. By mixing a large amount of ATH with the suspension of GNPs (ATH/GNP ratio of 100/0.5), the nanoplatelets were found to be well dispersed with ATH, indicating the positive effect of ATH in attenuating the aggregation of GNPs.

GNPs in PE/ATH/GNP composites contributed to the improved thermal stability. The temperature corresponding to 10% weight loss of PE/ATH/GNP (347 °C) was 4 °C higher than that of PE/ATH (343 °C). When the dehydration completed, PE/ATH/GNP showed better thermal stability. The temperature at 30% weight loss of PE/ATH/GNP was 3 °C higher than that of PE/ATH. Enhancement in the thermal stability in the presence of GNPs would be connected to the confined mobility of the polymer chains in a plane parallel to the confining graphene surfaces [39]. The formation of layered structure can act as a mass transport barrier to the degradation products, which also affected the onset of degradation.

Considering the well dispersed nanoplatelets in the composites, the formation of the char layers can be attributed to the immigration of GNPs to the surface during combustion [38]. Thus, GNPs were involved in the formation of the surface protective layers and contributed to the enhanced fire retardancy. Due to the well delaminated structure and uniform dispersion, GNPs can play a profound role in fire retardancy of PE/ATH at a loading as low as 0.2 wt %. A high aspect ratio of graphene was also reported responsible for the increase in barrier effect of the formed intumescent char [35]. According to the CONE results, GNPs were found to be effective in improving the barrier properties of residue of PE/ATH, leading to the improved fire safety of PE/ATH/GNP composite. 

Taking the low electrical percolation threshold (0.1 vol. %) of graphene into content [40], the low loading of GNPs will be helpful in keeping the insulation properties of PE/ATH/GNP composites. The enhancement of the fire-retardant properties of the composites with 0.2 wt % GNPs was impressive since only the well delaminated structure and good dispersion of GNPs could account for the interesting reduction.

## 5. Conclusions

GNPs with multi-layered structure and high structural integrity were prepared by fully exfoliated EG via ball milling coupled with ultrasonication. Delamination of EG in SDS solution was proven to be helpful in obtaining GNPs solution. By mixing ATH with GNPs suspension, the nanoplatelets were found to be well dispersed in ATH/GNP, indicating the positive effect of ATH in attenuating the aggregation of GNPs. 

PE/ATH/GNP had better thermal stability than PE/ATH while ATH/GNP initiated the decomposition at lower temperature. For the PE/ATH/GNP composite, when the dehydration of ATH completed, the char formed and acted as barrier to stabilize the PE chains and lead to the lower degradation rate at higher temperature. The enhanced fire retardancy of PE/ATH was revealed with addition of GNPs at a loading as low as 0.2 wt % by Cone testing. Comparing to PE/ATH, PE/ATH/GNP showed a reduced peak-HRR by 20%. A protective char layer composed of GNPs was evidenced to make the profound contribution to the enhanced fire retardancy. 

The well delaminated structure and good dispersion of GNPs accounted for the formation of effectively protective layer. The element analysis and morphological structure by XPS and SEM provided the evidences for the GNPs based char structure and the mechanistic barrier effect due to the introduction of GNPs. 

The method by compounding ATH with GNPs suspension would be a facile way to prepare the well dispersed ATH/GNP mixture as promising halogen-free flame retardant for industrial application of wire and cable.

## Figures and Tables

**Table 1:** TG data of ATH and ATH/GNP.

Sample	ATH	ATH/GNP
T_5_ (°C)	290	289
T_10_ (°C)	307	305
T_20_ (°C)	328	326
T_30_ (°C)	367	380
T_max_ (°C)	323	323
R_max_ (%/min)	10.28	10.06
Residue at 600 °C (%)	65.0	64.8

**Table 2:** TG data of PE/ATH and PE/ATH/GNP.

Sample	PE	PE/ATH	PE/ATH/GNP
T_5_ (°C)	445	321	328
T_10_ (°C)	461	343	347
T_20_ (°C)	473	465	468
T_30_ (°C)	478	482	485
T_max_ (°C)	492	499	502
R_max_ (%/min)	56.09	35.03	36.88
Residue at 600 °C (%)	0.0	24.7	25.5

**Table 3:** XPS data of char residue of PE/ATH and PE/ATH/GNP after Cone testing.

Sample	PE/ATH	PE/ATH/GNP
C:O	0.6	1.41
C:Al	1.41	4.08
Al:O	0.42	0.34
C1s Binding Energy (eV)	285.4	284.6
